# Kicking in the Guts: *Schistosoma mansoni* Digestive Tract Proteins are Potential Candidates for Vaccine Development

**DOI:** 10.3389/fimmu.2015.00022

**Published:** 2015-01-28

**Authors:** Barbara Castro-Pimentel Figueiredo, Natasha Delaqua Ricci, Natan Raimundo Gonçalves de Assis, Suellen Batistoni de Morais, Cristina Toscano Fonseca, Sergio Costa Oliveira

**Affiliations:** ^1^Laboratório de Imunologia de Doenças Infecciosas, Departamento de Bioquímica e Imunologia, Instituto de Ciências Biológicas, Universidade Federal de Minas Gerais, Belo Horizonte, Brazil; ^2^Instituto Nacional de Ciência e Tecnologia em Doenças Tropicais (INCT-DT), Conselho Nacional de Desenvolvimento Científico e Tecnológico, Ministério de Ciência Tecnologia e Inovação, Universidade Federal de Minas Gerais, Belo Horizonte, Brazil; ^3^Laboratório de Esquistossomose do Centro de Pesquisas René Rachou, Fundação Oswaldo Cruz, Belo Horizonte, Brazil

**Keywords:** *Schistosoma mansoni*, vaccine, digestive tract, esophageal gland, gastrodermis, proteases

## Abstract

Schistosomiasis is a debilitating disease that represents a major health problem in at least 74 tropical and subtropical countries. Current disease control strategies consist mainly of chemotherapy, which cannot prevent recurrent re-infection of people living in endemic area. In the last decades, many researchers made a remarkable effort in the search for an effective vaccine to provide long-term protection. Parasitic platyhelminthes of *Schistosoma* genus, which cause the disease, live in the blood vessels of definitive hosts where they are bathed in host blood for many years. Among the most promising molecules as vaccine candidates are the proteins present in the host–parasite interface, so numerous tegument antigens have been assessed and the achieved protection never got even close to 100%. Besides the tegument, the digestive tract is the other major site of host–parasite interface. Since parasites feed on blood, they need to swallow a considerable amount of blood for nutrient acquisition. Host blood ingested by schistosomes passes through the esophagus and reaches the gut where many peptidases catalyze the proteolysis of blood cells. Recent studies show the emergence of antigens related to the parasite blood feeding, such as esophageal gland proteins, proteases, and other proteins related to nutrient uptake. Herein, we review what is known about *Schistosoma mansoni* digestive tract proteins, emphasizing the ones described as potential vaccine candidates.

## Introduction

Schistosomiasis is considered the most important human helminth infection in terms of global mortality in developing countries. It is estimated that schistosomiasis causes 280,000 deaths in Sub-Saharan Africa alone (1, 2). The morbidity rates are also high and this disease is responsible for the loss of up to 4.5 million DALYs (disability adjusted life years) annually (3). Many countries have been investing in intervention strategies based on mass drug administration (4, 5). Nonetheless, the massive treatment has not decreased the endemicity due to constant re-infection of people and low quality of sanitary conditions in endemic areas (6–8) and yet, around 800 million people live in risk areas in 74 developing countries, of which at least 200 million people are infected (3, 9).

Many scientists search for a feasible vaccine against schistosomiasis; they believe this is the best strategy for reducing the disease transmission and morbidity. If a vaccine induced even a partial reduction in worm burdens it would considerably reduce pathology and limit parasite transmission (10, 11). Preventive vaccination would clearly overcome re-infection problems avoiding the need for repeated treatments of people living in endemic areas. As a result, vaccine strategies represent an essential component as an adjunct to chemotherapy for the control of schistosomiasis. The improvement in understanding of the immune response to schistosomes suggests that development of a vaccine is possible (5). In the search for vaccine targets, proteins located at the parasite/host interface are likely to be the most important, since they are commonly associated with mechanisms of escape from the host immune system or other adaptation to parasitism (12). The two major surfaces that constitute these interfaces are the outer tegument and the gastrodermis – gut lining (13). The tegument has been the major target of vaccine development. Recently, however, scientists are also focusing on schistosome gut and its secreted products. Protection studies demonstrate the potential of an esophageal gland secreted protein, gut proteases, and other gastrodermis proteins to be exploited for vaccine development. Throughout this work, we will review the digestive tract of *Schistosoma mansoni* and also present and discuss recent studies in vaccine development.

## Digestive Tract

The digestive tract of *S. mansoni* consists of the oral sucker, the esophagus, which is surrounded by the esophageal gland in its posterior portion and the blind-ended gut. Schistosome mouth is subapical and opens through the oral sucker, which is the beginning of the digestive tract in the anterior extremity of the worm. The oral sucker is a funnel-shaped vent covered by the tegument and bears thousands of spines, which extend up to the esophagus (14–16). The esophagus immediately succeeds the mouth opening and connects the sucker to the gut. It is a short tube that is invested by longitudinal and circular muscle fibers and two stronger circular muscle act as sphincters that control the entrance of blood (14, 17, 18). The esophagus is covered by an extension of the syncytial tegument with higher specialized surface architecture, specially focused in blood processing, and also an enormous membrane expanded into plates (17). The posterior half of the esophagus is surrounded by the esophageal gland that releases secretions into its lumen to process ingested host blood (17). The esophagus ends into the beginning of the gut. Then, posterior to the ventral sucker, the gut bifurcates into two lateral portions, which run either side of the reproductive organs and rejoin posterior to them, forming again a single portion that continues to the posterior end of the worm body. Since the gut is blind-ended, secretion of products might occur by regurgitation through the mouth (19, 20). The surface of the gut is a syncytial epithelial layer known as gastrodermis. Although gastrodermis is syncytial like the tegument, its cytoplasm differs by the presence of numerous mitochondria, nuclei, and biosynthetic machinery, with an active Golgi apparatus, a well-developed granular endoplasmic reticulum and numerous vesicles (19–21). Besides that, the gut syncytium is extended by the presence of fine cytoplasmic lamellae that characterize its absorptive nature (22, 23).

The primary function of the schistosome digestive tract is to digest macromolecules acquired from the blood of the mammalian host, and to absorb the soluble products (24). To live and reproduce inside the host, the mature, blood feeding worms use amino acids derived from degradation of serum proteins and lysed erythrocytes. Previous studies suggest that lysosomes directly secrete their contents into the gut lumen to digest incoming plasma (22, 24). The digestion process is presumed to be predominantly extracellular with the final stages possibly intracellular (22, 24, 25). Several peptidases are employed in processing and acquisition nutrients provided by host blood (26–28). Carrier proteins sequester essential organic and inorganic nutrients for uptake into the gastrodermis and the accumulated products are periodically eliminated from the blind-ending gut by regurgitation (22, 24). The structure of the gastrodermis has been well documented by transmission and scanning electron microscopic studies (19–21), but its protein composition has yet to be fully explored.

### Proteomic analyses

While the tegument has been the major target of characterization studies of *S. mansoni* for many years, the schistosome gastrodermis is a difficult target since it is inaccessible, what makes it pretty hard to collect samples. An alternative technique to have some evidences about digestive proteins is the collection of worms’ vomit, what is also difficult because the worms do not open their mouth easily in culture (22). The very first proteomic study on the vomit induced regurgitation in adult worms by osmotic shock with distilled water. Although this technique presents some contaminations from other tissues, it provides with at least an idea of gut contents. Some host proteins (hemoglobin, immunoglubolin (Ig)G, and serum albumin) were identified as well as worm antioxidants [superoxide dismutase (SOD) and thioredoxin] and proteins related to fatty acid absorption (lysophospholipase and fatty acid binding protein Sm14) (29). Biochemical analyses on vomit obtained either by osmotic or temperature shock demonstrated enzymatic activity, confirming that schistosomes utilize proteases to digest hemoglobin and host serum proteins (28, 30). Another proteomic study extended significantly the list of constituents secreted into the schistosome gut lumen as part of the blood feeding process. The authors induced the regurgitation by both temperature shock and protein starvation and the vomit contents were analyzed through electrophoresis followed by mass spectrometry. A total of 71 distinct proteins were identified, among them many proteases (cathepsins B1, C and S/L, asparaginyl peptidase and two proline carboxypeptidases, pro-X carboxylpeptidase, and dipeptidylpeptidase II), protease inhibitors (serpin and α2 macroglobulin), a series of lysosomal proteins (lipid-binding saposins and cholesterol-binding NPC-2), ion transporters (ferritins and calumenin), and also the previously identified antioxidants (SOD and thioredoxin) (24). The authors suggest that, due to the morphological changes in the gastrodermis and presence of cytoplasmic proteins (such as Sm14), parasite vomitus contents have contamination as a result of worm damage (24). Besides vomitus analyses, Nawaratna and colleagues (23) used a novel technique, the laser capture microdissection to recover the gut epithelium from tissue sections for analysis of gene expression by RT-PCR. This study identified 121 up-regulated transcripts, as expected, proteases (cathepsins A, B, D2, and L), lysosomal proteins (saposins, NPC-2, LAMP acid lipase, and phospholipase A2), transporters (Ca^2+^ ATPase, phospholipid transporter, and amino acid transporter), and the antioxidant SOD were identified, in agreement to proteomic investigations (23).

## Targeting the Gut

The relationship between the parasite and its host is largely nutritional, involving the unidirectional transfer of nutrients. In this context, schistosome seeking for food has been the primary selection pressure to initiate the evolution of host–parasite association (31, 32). Schistosomes not only feed on blood, but are bathed in their food. Adult male *S. mansoni* is estimated to ingest some 39,000 erythrocytes hourly, while the female, due to egg production, requires 10 times more, 330,000 erythrocytes hourly (33). In *S. mansoni*, the lysis of blood cells takes place exclusively in the digestive tract; it begins in the esophagus, where the esophageal gland secrets digestive enzymes and erythrocytes are rapidly lysed (15, 17). Then, peristaltic movements pass lysed cells down into the anterior gut and, finally the gut lumen takes up the essential nutrients (20).

Blood is essential to worm survival since it provides a consistent and renewable nutrient resource (31). Eventual impairments to blood processing would compromise parasite development inside the host leading them to death through starvation. There are at least two evidences showing this phenomenon. First, the treatment of infected mice with cysteine protease inhibitors not only reduces significantly worm burden but it also inhibits egg production by females. Authors believe that inhibitors arrest hemoglobin degradation causing obstruction of the feeding activity and nutrient uptake (34, 35). Besides, the elimination of *S. mansoni* adult worms by rhesus macaques seems to be related to gut damage. Surviving moribund worms had alterations in their intestinal epithelium and absence of food in the gut lumen, pointing to worm starvation. The authors suggest that blocking antibodies impact on nutrient uptake by both gut and tegument (36). In this context, interventions that block blood processing reveal new anti-schistosome targets for schistosomiasis control and so, the digestive tract and its secretions appear to be a great source of key antigens.

## Esophageal Gland and Sm10.3 as Vaccine Candidate

The esophageal gland is a bi-lobed structure that lies around the posterior esophagus. The first morphology study on the esophageal gland, in the late 1970s, suggested its function in initial blood digestion (15). Recently, a detailed morphological and functional study on this gland confirmed the central role of esophagus in blood processing, not acting simply as a conduit (17). The architecture of the esophageal gland comprises as many as 1000 cell bodies, each connected to posterior esophagus by microtubule-lined extensions. Cell bodies are specialized in protein production and export, they synthesize large numbers of crystalloid vesicles and release their contents into the lumen (17, 37). Esophageal gland secretions are responsible, among other activities, for initiating hemolysis and blood digestion. Erythrocytes are lysed upon their entry to the posterior esophagus and very few intact cells are seen beyond that point (17, 24). Besides, these secretions may also act neutralizing host immune effectors and contributing to parasite survival. Leukocytes are somehow tethered in the posterior lumen in variable states of degeneration (13, 17, 19).

Micro exon gene (MEG) proteins 4.1, 4.2, and 14 (38) and also venom allergen-like (VAL) protein 7 (39) are some of the proteins identified in the esophageal gland (17). Recent study demonstrated preliminary evidence that MEG-4.1, also termed Sm10.3, induces erythrocyte agglutination *in vitro*, what could be related to erythrocyte digestion (40). Besides proteomic studies, a transcriptional analysis of esophageal gland revealed that more than 120 genes are differentially up-regulated there. The great majority of transcripts identified code for proteins related to binding or hydrolase activities, which may be important to nutrient uptaking and red cell lysis and catabolism (41).

Sm10.3 antigen was used in vaccine development against schistosomiasis in the murine model. When formulated with Freund’s adjuvant, this antigen induced a mixed Th1/Th2-type response, as IFN-γ, TNF-α, and low levels of IL-5 were detected in the supernatant of cultured splenocytes (40). The vaccination also reduced in 32% the worm burden and ameliorated liver pathology, since 43.6% less eggs were found in the liver and there was a significant reduction in the number, size and fibrosis of granulomas, 23.8, 11.8, and 39.8%, respectively. This data suggest that Sm10.3 is a potential candidate for vaccine development (40). These findings, together with the detection of host IgG binding in schistosome esophagus, suggest that esophageal gland proteins are attractive vaccine candidates, not only because they mediate initial feeding process, but also because antibodies targeting such proteins would not face the hostile acid proteolytic environment in the gut (17).

## Gut Proteases

Schistosome proteases are involved in a vast range of essential processes such as invasion, migration, feeding, reproduction, activation, and evasion of immune system [reviewed in Ref. (26, 42)]. Genomic studies identified at least 250 peptidases in the genome of *S. mansoni* (43, 44). In the feeding context, many proteases have been identified in the regurgitated gut contents by biochemical and proteomic analysis (24, 30). In addition, several studies confirm the presence of proteases in the gastrodermis by immunocytochemistry and point out the roles of cysteine endopeptidases, cathepsin L, cathepsin D, cathepsin B, asparaginyl endopeptidase, and metalloproteases in hemoglobin and serum albumin degradation and processing (45–48). The great amount of proteases in the gut provides significant redundancy in blood protein degradation (27). Since *S. mansoni* gut proteases have a vital role in blood processing researchers believe that they are promising vaccine candidates and some of them have already been tested (26, 42, 49, 50).

*Schistosoma mansoni* cathepsin-B1 (SmCB1) is the most abundant papain-like cysteine peptidase in the parasite gastrodermis (51), it was first localized in the parasite gut lumen, however, another study hypothesized that this protein could be also expressed in the cecum and protonephridia of cercariae (52). Immunological studies with schistosomiasis patients suggested that SmCB1 is an immunodominant target of the immune response during pre-patent schistosome infection since authors demonstrated that SmCB1 is targeted by IgG and IgE specific antibodies (53). El Ridi and colleagues (50) evaluated the potential of active SmCB1 as vaccine with its inbuilt adjuvanticity or as adjuvant to another known candidates (54). In the first situation, SmCB1 reduces significantly worm burden (66–73%), eggs in liver (51%), and in small intestine (25%). However, when SmCB1 is incubated with proteinase-inhibitor prior to immunization, the levels of protection decrease significantly, pointing out the importance of the peptidase activity in protective potential. The immunization with active form of SmCB1 by itself also induces the production of high levels of IL-4, IL-5, and IL-13 and high titers of IgG, IgG1, and IgG2b (54). Besides being a promising vaccine target, SmCB1 also acts as an adjuvant, since it increases the protection provided by immunization with other proteins, such as glyceraldehyde 3-phosphate dehydrogenase and peroxiredoxin-multiple antigen peptide, from <10%, formulated in Th1 adjuvants, to up to 84%, formulated with SmCB1 (54, 55).

The protein Sm32 is an asparaginyl peptidase (SmAE) member of the legumain family that, probably, cleaves zymogens of proteinases involved in hemoglobin degradation (56–58). Its activation is an autocatalytic event and it is related to a loss of a C-terminal portion and an N-terminal pro-domain, what reduces the protein from 50 kDa to approximately 32 kDa (59). Several peptides of SmAE were chemically synthesized and, when administrated in a Freund’s formulation, showed relative immunogenicity in rabbits and mice, in particular the hydrophilic regions of the molecule (56, 57). When SmAE was evaluated as DNA vaccine, it elicited a significant humoral response and egg count reduction (32%), but it failed to reduce the entire worm burden (60).

Cathepsin D (SmCD), an aspartic protease, seems to be involved in hemoglobin process in *S. mansoni* (28, 61). Schistosomula treated with RNAi to SmCD were unable to develop and survive in mice, indicating the crucial functions of this protease in parasite maintenance (48). Two SmCD peptides formulated with lipid core scaffold (LCS) elicit a humoral response in mice. The anti-SmCD antibodies recognize the native SmCD in adult worms protein extracts, and almost abrogate its enzymatic activity *in vitro*, pointing out the potential of this protein as a vaccine target (62).

## Other Gut Proteins as Vaccine Targets

### Superoxide dismutase

Immunolocalization assays demonstrated the antioxidant enzyme Cu/Zn SOD in the two major host–parasite interfaces: schistosome tegument and gastrodermis. The localization of this protein suggests the development of parasite antioxidant mechanisms to protect themselves against the host cellular response and also against hemoglobin oxidation products, respectively (63). Schistosome SOD was also detected in proteomic analyses of worm’s vomit and transcriptional analysis of digestive tract (23, 24, 29). DNA vaccination strategies using Cu-Zn SOD cDNA protected mice from *S. mansoni* infection reducing the worm burden up to 54%; two SOD peptides were also tested and reduced worm burden from 31 to 51% (64). In another vaccination experiment, one SOD peptide, CT-SOD, also formulated as DNA vaccine, induced a Th1-immune response and significantly decreased worm burden (36–43%) after surgical transfer of adult worms into the mesenteric veins of immunized mice (65). A cross-reactivity study demonstrated that mice anti-SmSOD antibodies or infected individual sera do not recognize human SOD in its native form, meaning that SmSOD could serve as a basis for developing a vaccine against schistosomiasis (66).

### Syntenin

In a recent study, schistosome syntenin (SmSynt), a PDZ-domain protein, was localized in the gastrodermis of *S. mansoni*, even though none of the proteomic and transcriptional studies identified this protein at this location (67). The role the protein plays in the natural biology of the parasites remains unclear since knocking down the expression of the SmSynt gene yielded no clear phenotype *in vitro*. However, syntenin is involved in many cellular processes in mammalian cells, among them, cellular trafficking and biogenesis of small extracellular vesicles, what could be also its function in schistosome digestive tract. The recombinant SmSynt seems to be a relevant vaccine target since it induces the production of IgG antibodies and Th1-cytokines likely important in disease control. Mice vaccinated with the recombinant protein induced 30–37% of worm burden reduction. A reduction in liver damage was also noted in vaccinated versus control mice, 38–43% reduction in the number, and 35–37% reduction in the area of liver granulomas (67).

### Saposin

Four proteins possessing the characteristic saposin domain were identified in schistosome vomit (24). Saposins bind sphingolipids, facilitating their degradation by ceramidases and they also bind other lipids, sequestering them in the gut lumen for transport/uptake into the cells (24, 68). A gut saposin-like protein (SmSLP-1) has proven to be immunogenic since antibodies from infected individuals recognized its recombinant form, however, it was ineffective as a vaccine in the mouse model. SmSLP-1 formulated with Freund’s adjuvant was utilized in a vaccination trial, and despite the presence of high antibody titers in immunized mice, adult worms, and eggs recovered from vaccinated animals were the same as in control group (68). Unfortunately, not all gut proteins tested as vaccine were successful.

## Concluding Remarks and Future Perspectives

**Figure 1:**
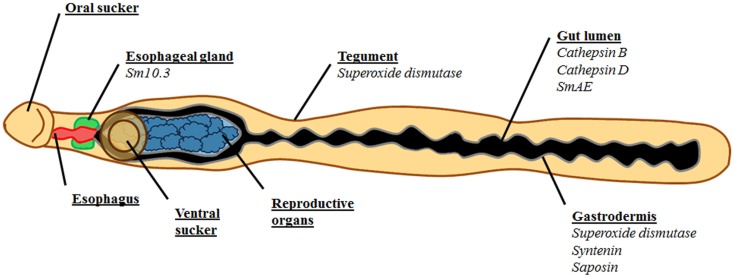
**Schematic representation of *S. mansoni* emphasizing the digestive tract and its antigens tested as vaccine**. Esophagus is represented in red, esophageal glands are green, gut lumen is black, and gastrodermis is gray. The reproductive organs, which are surrounded by the bifurcated gut, are represented in blue. The tegument is brown, as well as the oral and ventral suckers. The antigens are listed under the location they were identified in italics. Superoxide dismutase was identified both in the tegument and in the digestive tract.

Hematophagous parasite *S. mansoni* succeeds in the tough job of surviving inside human host and this success can be attributed, among other abilities, to their capacity of actively feed via the digestive tract. Blood processing and nutrient uptake are critical for the survival of schistosomes and direct or indirect interruption of these processes may represent a realistic strategy for vaccine development. These interventions would probably lead worms to starvation, and consequently death, since insufficient supply of energy impairs growth, pairing, maturation, and fecundity. Here, we described the physiological function of *S. mansoni* alimentary tract and also reported some examples of successful vaccines formulated with digestive tract proteins, summarized in Table 1 and Figure 1.

**Table 1:** **Digestive tract antigens**.

Localization	Antigen	Vaccine formulation	Functions likely affected by vaccination	Worm burden reduction
Esophageal gland	Sm10.3	Recombinant protein	Initial blood processing	32%
Gut lumen	Cathepsin B	Recombinant protein	Hemoglobin processing	Up to 84%
	SmAE	DNA vaccine	Hemoglobin processing	None^a^
	Cathepsin D	Synthetic peptides	Apical processing of hemoglobin	–
Gastrodermis	Superoxide dismutase^b^	DNA vaccine	Antioxidant mechanisms; protection from hemoglobin oxidation products	Up to 54%
	Syntenin	Recombinant protein	Cellular trafficking and biogenesis of small extracellular vesicles	Up to 37%
	Saposin	Recombinant protein	Lipid binding, transport, and uptake	None

*^a^32% reduction in recovered eggs*.

*^b^Also identified in the tegument*.

The efficacy of these vaccines seems to remain on the generation of antibodies that are able to bind to the target enzyme and inhibit its enzymatic function in the parasites, compromising essential aspects of their biology. The gut lumen seems a hostile site for antibodies due to its low pH and the presence of multiples proteases. These features are probably the reasons why not all antigens conferred protection. However, despite the aggressive environment of the gut, immunization experiments showed that targeting molecules from digestive tract successfully yielded some protection against schistosomes. We are still in the beginning of understanding the complex feeding mechanisms by which schistosomes obtain nutrients from their host and we have no clue if antibodies are able to affect this process. However, we do know that infected patients generate high levels of antibodies against the gut secreted polysaccharide antigens circulating anodic and cathodic antigens (CCA and CAA), used in diagnostic tests (69), and also against the gut protease SmCB1 (53). These data indicate that gut antigens are somehow available to antigen presentation.

Proteomic analyses on schistosomes gut secretions revealed many cathepsins and other peptidases involved in the proteolytic pathways of host nutrients. The proteolytic pathways for hemoglobin and albumin have been described and they showed that a large number of proteases provide some redundancy (28). Functional redundancy is an issue for the development of vaccines based on gut proteases; however, this issue could be amended by formulating a vaccine comprising either a combination of proteases or a chimeric antigen consisting of epitopes of different enzymes (26). The better understanding of nutrient uptake and the advancement of new vaccination approaches are key points for the development of intervention strategies, specially the development of a vaccine.

## Conflict of Interest Statement

The authors declare that the research was conducted in the absence of any commercial or financial relationships that could be construed as a potential conflict of interest.
